# An unusual case of persistent consolidation: Idiopathic lymphoid interstitial pneumonia

**DOI:** 10.1002/rcr2.1408

**Published:** 2024-06-10

**Authors:** Harriet J. Caterson, Sewon Kim, Matthew Zaborowski, Michael Harden, Michael Hibbert

**Affiliations:** ^1^ Department of Respiratory Medicine Royal North Shore Hospital St Leonards New South Wales Australia; ^2^ Department of Anatomical Pathology Royal North Shore Hospital St Leonards New South Wales Australia; ^3^ Department of Cardiothoracics Royal North Shore Hospital St Leonards New South Wales Australia; ^4^ Northern Clinical School University of Sydney St Leonards New South Wales Australia

**Keywords:** interstitial, lung diseases, lymphoproliferative disorders

## Abstract

Lymphocytic interstitial pneumonia (LIP) is a rare but largely benign interstitial lung disease, most frequently associated with HIV and autoimmune conditions. It is infrequently found to be an idiopathic condition. Diagnosis is complex and can require numerous invasive tests as evidenced in the case presented. The diagnosis is made from a combination of clinical, radiological, and histological features but the unusual radiological and clinical features meant diagnosis in our case required surgical biopsy. There is minimal evidence around best treatment although largely involves targeting the underlying cause. There is a small risk of transformation to lymphoma and fibrosis. Immunosuppression with steroids is the most common therapeutic strategy however in our case the radiographic changes spontaneously resolved. We present a case of an immunocompetent male presenting with significant radiological and histopathological findings of LIP, without significant symptomatology, that spontaneously resolved without intervention suggesting a monitoring approach may be a valid management strategy.

## Graphical abstract

An unusual case of persistent consolidation in a gentleman requiring significant invasive and radiological investigation resulting in a rare diagnosis of an interstitial lung disease. Optimal management strategies are unclear and this case highlights the potential of close monitoring rather than immunosuppression for these patients.

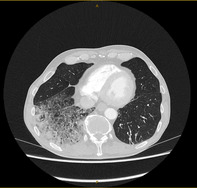

## INTRODUCTION

Lymphoid interstitial pneumonia (LIP) is a rare interstitial lung disease (ILD) most associated with Sjogren's syndrome and human immunodeficiency virus infection (HIV). LIP is characterized by infiltration of lymphoid cells and other cellular elements into the lung interstitium.^1^ Most cases of LIP are benign, but approximately 5% can transform into lymphomas.^2^ Definitive diagnosis is based on a combination of history, radiologic features, and a confirmatory biopsy. Few patients recover without therapy, whilst others progress to fibrosis or malignancy despite treatment.^3^ High resolution computed tomography (CT) imaging classically demonstrates thin‐walled cysts, centrilobular nodules and diffuse ground glass changes.^4^ Definitive diagnosis may be difficult, and treatment generally includes a trial of steroids or immunotherapy.

## CASE REPORT

A 66‐year‐old Caucasian male with a 45‐pack year smoking history presented with 6 months of chronic cough with two chest computed tomography (CT) scans showing persistent extensive right lower lobe consolidation. His cough had improved with two courses of oral antibiotics, without radiological amelioration. The patient's background included a tonsillar squamous cell carcinoma treated with tonsillectomy and definitive chemoradiotherapy. He was awaiting a partial cystectomy for a new diagnosis of urothelial malignancy. The patient had no significant respiratory, autoimmune or immunodeficiency diagnoses prior to presentation nor any relevant family history. He had occupational exposure to silica and asbestos, but below recommended workplace limits.

Chest auscultation revealed fine crackles in the right lung base. Otherwise, his physical examination was entirely normal. Sputum cultures and cytology were non‐diagnostic. Pulmonary function tests revealed moderate obstructive ventilatory impairment pattern with FEV1/FVC of 48.5% in keeping with the patient's diagnosis of chronic obstructive pulmonary disease. The patient's FEV1 measured 1.61L (47% predicted) and FVC 3.33L (74% predicted). There was no restriction with TLC 6.15L (83% predicted) and there was severely reduced diffusion capacity with DLcO 12.4 (predicted 46%).

**FIGURE 1:**
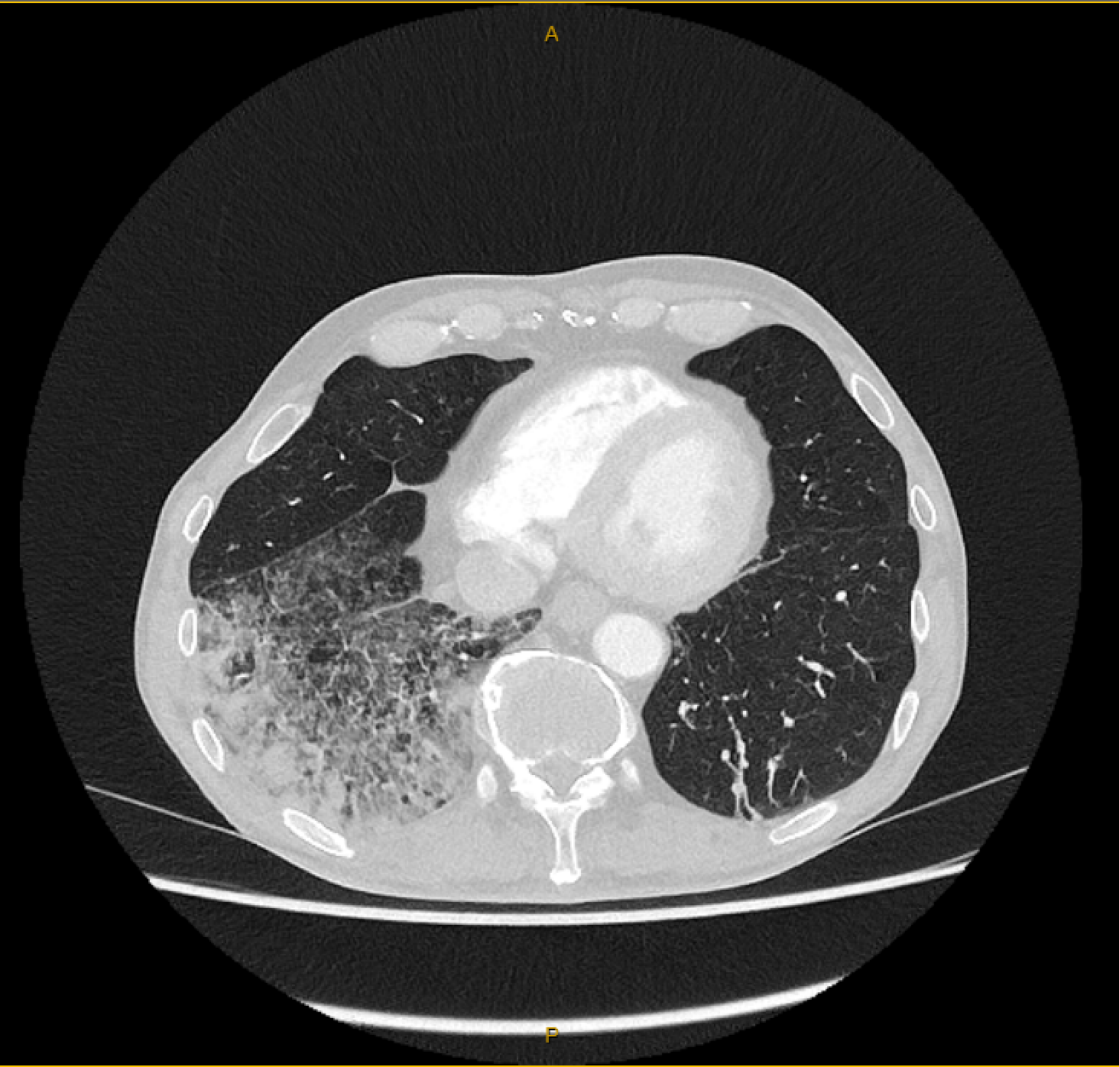
Axial computed tomography (CT) Chest on presentation demonstrating airspace consolidation and right lower lobe reticular shadowing.

Chest CT demonstrated widespread airspace consolidation in the right lower lobe with underlying reticular shadowing with mild mediastinal and right hilar lymphadenopathy (Figure 1). Concerned that this might represent a lung cancer with lepidic growth, an FDG PET scan was performed demonstrating mild FDG avidity in the right lower lobe with moderate avidity in bilateral mediastinal lymph nodes (SUV 6.3) and no abnormal avidity elsewhere. Bronchial washings of the right lower lobe revealed a mix of bronchial, squamous cells and lymphocytes with no malignant cells. A cellular differential was not obtained. Endobronchial Ultrasound/Transbronchial needle aspirate (EBUS/TBNA) of lymph node stations 4R and 7 demonstrated lymphocytes without malignancy. Flow cytometry on the TBNA sample demonstrated polyclonal B cells with normal kappa: lambda ratio. EBUS/TBNA and BAL were repeated on two occasions given ongoing concern for malignancy. A fine needle aspiration and core lung biopsy were also non‐diagnostic.

The patient was treated with 1 month of 25 mg of Prednisone for possible organizing pneumonia, without radiological improvement. A surgical lung biopsy was then performed. Intraoperative findings were of yellow, gelatinous material within the right lower lobe and haemorrhagic change on the pleural surface.

**FIGURE 2:**
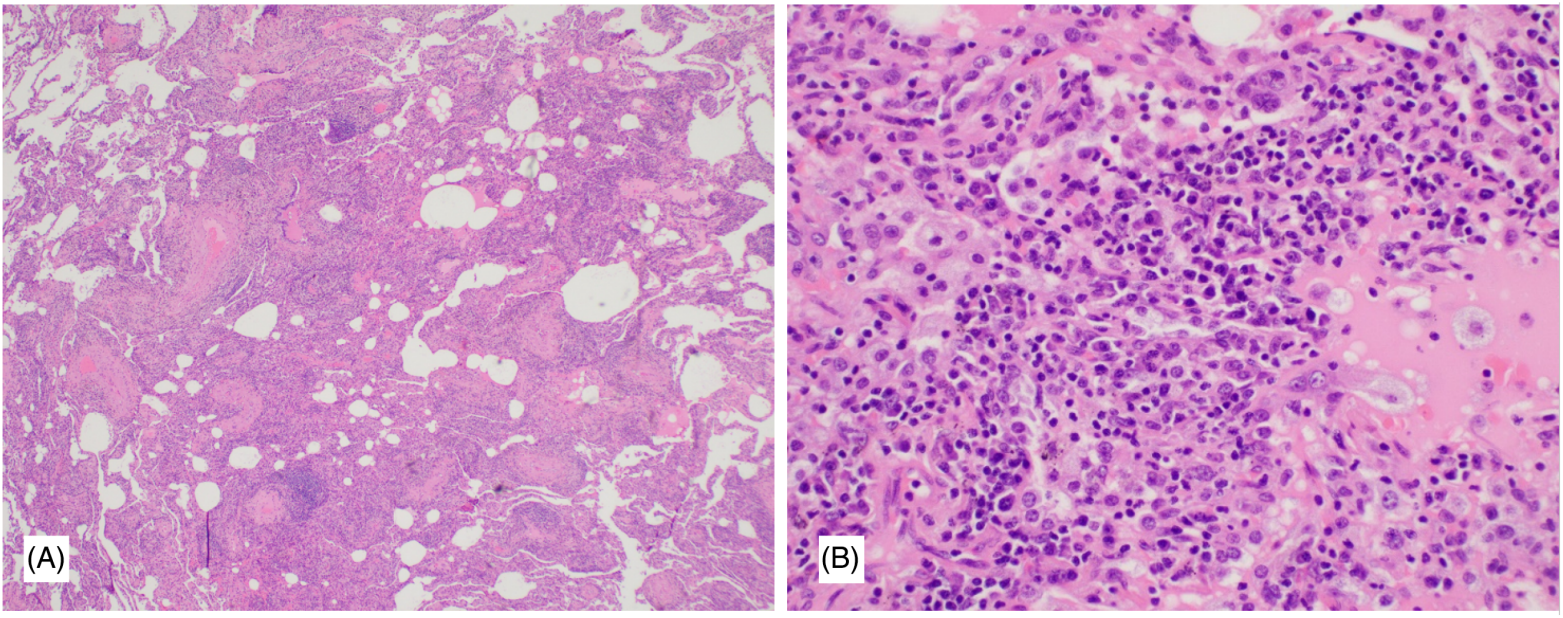
(A) Low power of LIP with diffuse chronic inflammatory infiltration into the intersitium, perivascular and peribronchiolar areas and fluid filled alveolar spaces. (B) Higher magnification showing lymphoplasmacytic infiltrate.

Histology showed a diffuse lymphoplasmacytic infiltrate in the interstitium, perivascular and peribronchiolar spaces with occasional lymphoid aggregates. There were rare giant cells present with a few isolated poorly formed granulomas including some with central necrosis in dilated lymphatic spaces. No fungal organisms or viral inclusions were detected on special stains including for HHV8, EBV and CMV. Immunohistochemistry showed a mixed T‐cell and B‐cell infiltrate and no evidence of monoclonality. A diagnosis of lymphoid interstitial pneumonia (LIP) was made after discussion in a multidisciplinary ILD meeting given the unusual findings (Figure 2).

Laboratory data showed normal C‐reactive protein (14 mg/dL), total leucocyte count (8900/mm^3^) without eosinophilia. HIV antigen and antibodies, antineutrophil cytoplasmic antibodies (ANCA), extractable‐nuclear antigens (ENA), anti‐nuclear antibody (ANA), double‐strand DNA (dsDNA), complement and immunoglobulin levels were also normal.

**FIGURE 3:**
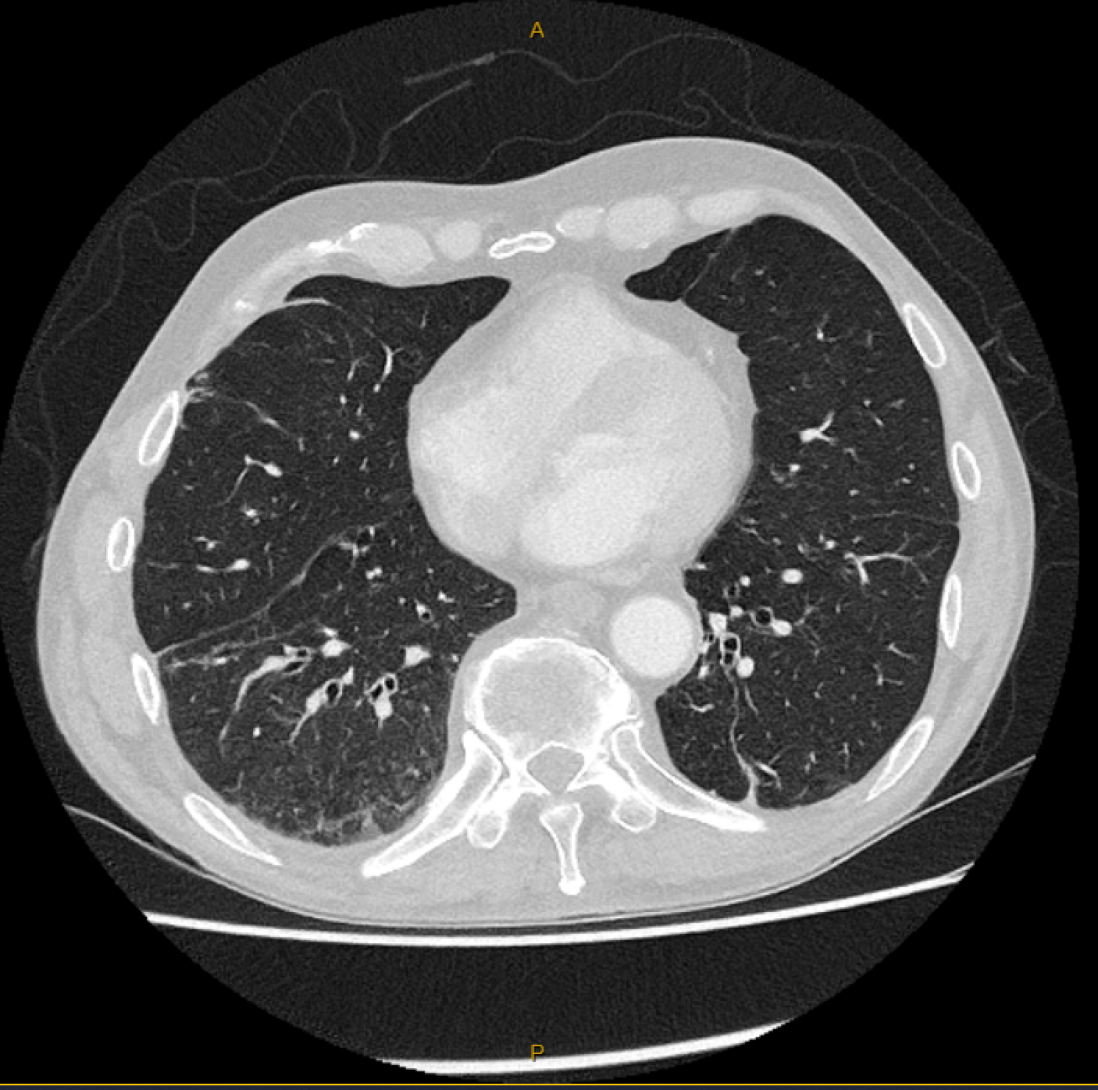
Axial computed tomography (CT) Chest after 6 months demonstrating resolution of majority of earlier imaging findings.

The patient remained minimally symptomatic and successfully underwent his urological procedure given no concern for lung malignancy was identified. After 6 months, repeat chest CT imaging demonstrated near complete resolution of imaging findings without specific therapy (Figure 3).

## DISCUSSION

Lymphoid Interstitial Pneumonia (LIP) is an uncommon ILD believed to occur in response to a range of stimuli. In the largest prospective case series of patients reviewing 15 subjects with LIP, the majority were female (*n* = 11) and had Sjogren's syndrome (*n* = 8).^2^ Studies indicate no underlying cause is found in less than 20% of cases. There is limited data around LIP's natural history and effective therapeutic agents. In patients with autoimmune and HIV related LIP, treatment of the underlying condition may help, although there are no controlled trials of any therapy.^3^ Corticosteroid treatment with or without immunosuppressive agents have been used; however, one‐third of patients may have an unfavourable clinical outcome ranging from pulmonary fibrosis to transformation to lymphoma.^5^ There are some reports of spontaneous resolution of LIP without treatment.^1^, ^6^

Our case is unusual as the patient was male, with no underlying immunodeficiency or connective tissue disease and was relatively asymptomatic with only mild cough. His radiology was not typical, showing dense lobar consolidation rather than ground glass with cystic change.^4^ This case also highlights the difficult diagnostic process associated with LIP, with our patient requiring several invasive procedures.

The histopathologic differential includes a cellular non‐specific interstitial pneumonia (NSIP) given many morphologic features overlap, as well as infectious and immunologic processes. In our case, there was dense lymphocytic infiltration in the interstitium, perivascular and peribronchiolar spaces, and not confined to infiltration in alveolar walls, as is seen in NSIP. It is also important to exclude viral pneumonia, such as EBV, and fungal pneumonitis. In this case, there were no viral inclusions or fungal elements seen on stains. Histopathologic findings are essential in the diagnosis of LIP and must be correlated with clinical and radiological findings.

While other cases of idiopathic LIP have been described, spontaneous resolution without treatment such as in our case is uncommon. Although there was resolution in conjunction with his partial cystectomy, there is no evidence of LIP representing a paraneoplastic phenomenon of urothelial cancer, or other cancers, upon our current literature review. An argument could thus be made in similar patients with few symptoms, for a period of careful monitoring rather than active therapy. Despite marked radiological improvement to this point, this patient's outlook remains uncertain. We plan to closely monitor him for radiological or symptomatic recurrence.

We present a rare case of LIP with classical histopathological findings but unusual clinical and radiological features, including spontaneous radiological resolution.

## AUTHOR CONTRIBUTIONS

*Conceptualization*: Michael Hibbert. *Interpretation of imaging findings*: Michael Hibbert, Michael Harden, Harriet Caterson. *Writing of the manuscript*: Harriet Caterson, Sewon Kim and Michael Hibbert. Interpretation of pathology findings: Matthew Zaborowski and Sewon Kim. *Editing of the manuscript*: Harriet Caterson, Sewon Kim, Michael Hibbert. All the authors have read and agreed to the published version of the manuscript.

## CONFLICT OF INTEREST STATEMENT

None declared.

## ETHICS STATEMENT

The authors declare that appropriate written informed consent was obtained for the publication of this manuscript and accompanying images.

## Data Availability

The data that support the findings of this study are available from the corresponding author upon reasonable request.

## References

[1] Swigris JJ , Berry GJ , Raffin TA , Kuschner WG . Lymphoid interstitial pneumonia: a narrative review. Chest. 2002;122(6):2150–2164. 10.1378/chest.122.6.2150 12475860

[2] Cha S‐I , Fessler MB , Cool CD , Schwarz MI , Brown KK . Lymphoid interstitial pneumonia: clinical features, associations and prognosis. Eur Respir J. 2006;28(2):364–369. 10.1183/09031936.06.00076705 16571614

[3] Garcia D , Young L . Lymphocytic interstitial pneumonia as a manifestation of SLE and secondary Sjogren's syndrome. BMJ Case Rep. 2013;2(2013):bcr2013009598. 10.1136/bcr-2013-009598 PMC3762176 23912652

[4] Louza GF , Nobre LF , Mançano AD , Hochhegger B , Souza AS Jr , Zanetti G , et al. Lymphocytic interstitial pneumonia: computed tomography findings in 36 patients. Radiol Bras. 2020;53(5):287–292. 10.1590/0100-3984.2019.0107 33071371 PMC7545736

[5] Arcadu A , Moua T , Yi ES , Ryu JH . Lymphoid interstitial pneumonia and other benign lymphoid disorders. Semin Respir Crit Care Med. 2016;37(3):406–420. 10.1055/s-0036-1580691 27231864

[6] Kokosi MA , Nicholson AG , Hansell DM , Wells AU . Rare idiopathic interstitial pneumonias: LIP and PPFE and rare histologic patterns of interstitial pneumonias: AFOP and BPIP. Respirology. 2016;21:600–614. 10.1111/resp.12693 26627191

